# Evaluating the effectiveness of clinical ethics committees: a systematic review

**DOI:** 10.1007/s11019-020-09986-9

**Published:** 2020-11-21

**Authors:** Chiara Crico, Virginia Sanchini, Paolo Giovanni Casali, Gabriella Pravettoni

**Affiliations:** 1 Department of Oncology and Hemato-Oncology, University of Milan, Milan, Italy; 2 Applied Research Division for Cognitive and Psychological Science, IEO, European Institute of Oncology IRCCS, Milan, Italy; 3 Department of Public Health and Primary Care, Centre for Biomedical Ethics and Law, KU Leuven, Leuven, Belgium; 4 Fondazione IRCCS Istituto Nazionale Tumori, Milan, Italy

**Keywords:** Clinical ethics committees, Ethics committees, Systematic review, Effectiveness, Evaluation, Satisfaction, Helpfulness

## Abstract

Clinical Ethics Committees (CECs), as distinct from Research Ethics Committees, were originally established with the aim of supporting healthcare professionals in managing controversial clinical ethical issues. However, it is still unclear whether they manage to accomplish this task and what is their impact on clinical practice. This systematic review aims to collect available assessments of CECs’ performance as reported in literature, in order to evaluate CECs’ effectiveness. We retrieved all literature published up to November 2019 in six databases (PubMed, Ovid MEDLINE, Scopus, Philosopher’s Index, Embase and Web of Science), following PRISMA guidelines. We included only articles specifically addressing CECs and providing any form of CECs performance assessment. Twenty-nine articles were included. Ethics consultation was the most evaluated of CECs’ functions. We did not find standardized tools for measuring CECs’ efficacy, but 33% of studies considered “user satisfaction” as an indicator, with 94% of them reporting an average positive perception of CECs’ impact. Changes in patient treatment and a decrease of moral distress in health personnel were reported as additional outcomes of ethics consultation. The highly diverse ways by which CECs carry out their activities make CECs’ evaluation difficult. The adoption of shared criteria would be desirable to provide a reliable answer to the question about their effectiveness. Nonetheless, in general both users and providers consider CECs as helpful, relevant to their work, able to improve the quality of care. Their main function is ethics consultation, while less attention seems to be devoted to bioethics education and policy formation.

## Background

Clinical Ethics Committees (CECs) or Hospital Ethics Committees are bodies originally established with the aim of supporting healthcare professionals in managing controversial ethical issues affecting clinical practice (Fleetwood et al. 1989) that cannot be settled simply in terms of medical competence (Renzi et al. 2016). The same aim is pursued by all those services commonly labelled as Clinical Ethics Support Services (CESS), i.e. services aiming at supporting health-care professionals and/or patients and their relatives in dealing with health-related clinical ethics issues. Clinical Ethics Committees represent one of the most common explicit forms of CESS, together with facilitation of Moral Case Deliberation (MCD) and individual ethics consultants (Molewijk et al. 2015).

CECs deliver ethics support in many ways, by undertaking a variety of tasks that, over time, scientific literature has categorized as follows: ethics consultation, policies formation and/or revision, and bioethics education (Aulisio and Arnold 2008). Although CECs developed in parallel with Research Ethics Committees (RECs) (or Institutional Review Boards, as labelled in the US), CECs are much less enforced and their tasks are much less harmonized.

Since their first appearance in the late 1970, CECs and the other forms of ethical support services have grown up widely in the United States (Saunders 2004): data by McGee and colleagues report that 93% of the hospital sampled had a CEC by the year 1999 (McGee et al. 2001); Fox and colleagues on a predictive sample basis, estimate that 80% of US hospitals have some forms of ethical support service, with a further 14% in the process of developing one (Fox et al. 2007). CECs, and CESS more in general, are increasingly developing also in healthcare institutions in the rest of the world (Dörries et al. 2011; Hurst et al. 2007), with differences with respect to their diffusion (Fox et al. 2007; Hajibabaee et al. 2016; Slowther et al. 2001), internal structure, largely depending on the local culture and context (Czarkowski et al. 2015; Guerrier 2006; Hurst et al. 2007; Meulenbergs et al. 2005; Pitskhelauri 2018; Zhou et al. 2009), functions (Hajibabaee et al. 2016), and model of CESS delivering (Molewijk et al. 2015).

Although the number of publications concerning CECs is high, their actual effectiveness in clinical practice has yet to be clarified. As a matter of fact, CECs are generally the most common model of CESS in many countries (Molewijk et al. 2015), but the latest literature investigating performance evaluation focuses more on other forms of CESS (Chen et al. 2014; Haan et al. 2018) or CESS in general, including some recent literature reviews (Haltaufderheide et al. 2020; Hem et al. 2015). Indeed, most of the scientific literature on CECs either reports the history of their development (Courtwright and Jurchak 2016; Saunders 2004), or describes their activities and functions (Rasoal et al. 2017), or provides theoretical contributions about their role in hospitals and care centers (Fleetwood et al. 1989; Jansen et al. 2018). Therefore, despite having been established in order to address the need for an ethics discussion on controversial and morally sensitive clinical cases, it is still unclear whether and to what extent they managed to accomplish this task.

Contrary to what one may expect, this is not a recent question, as the need for a thorough evaluation of CECs’ performance was recognized early in the formation of these committees (Griener and Storch 1992; Lo 1987). After more than 40 years since their beginnings, the matter is still unclear and studies investigating how CECs actually affect healthcare are still limited. As a consequence of the dearth of evidence available about their effectiveness (Slowther et al. 2002), some authors challenged the need to establish CECs (Fletcher and Hoffmann 1994; Hipps 1992; Williamson et al. 2007), especially from a cost–benefit perspective.

Nowadays, the question around CECs’ effectiveness is even more relevant, since many countries have only recently started to implement CESS in their different forms (Hajibabaee et al. 2016). In particular, in those countries where no specific founds are appointed for this function, ethics support services are delivered either by RECs or by CECs, developed following RECs’ institutional framings (Slowther and Hope 2000).

Drawing from these premises, the overarching aim of this systematic review is to gain a comprehensive overview on the *assessed effectiveness of CECs*, both interpreted as *subjective* outcome, namely the index of how the stakeholders who benefited from CECs experienced it, and as *objective* outcome, that is, the tangible consequence of CECs’ activities, measurable in daily clinical practice (e.g., as a change in the management of patient care path).

By collecting and clarifying evaluation tools used to assess the effectiveness of CECs in healthcare, we also try to answer the question whether CECs are useful resources.

## Methods

### Search strategy

A large number of studies refer to ethics committees as broadly conceived, thus including both CECs and RECs. Therefore, the search string had to be narrowed down in order to include only ethics committees devoted to clinical practice. The search focus was represented by CECs. Furthermore, all the possible definitions of CECs had to be taken into account: *clinical ethics committees*, *hospital ethics committees*. Therefore, as terms and/or keywords (e.g., mesh terms) all the expressions referring to—or containing under their trees—the aforementioned terms were included.

On these premises, the string was built in relation to two semantic groups: group A included all possible definitions and mesh terms related to CECs; group B contained all terms pertaining to assessment, impact, and/or evaluation. In particular, group A contained the following terms: Clinical ethics committee*, hospital ethics committee*; while group B contained: impact, effectiveness, evaluation*, assessment*.

The two groups were then gathered according to the properties and Boolean operators of each database (see Table 1). The choice of the terms as well as the search strings were developed by the first author (CC) in consultation with the second author (VS). In order to cover both the fields of healthcare science, clinical/medical ethics and bioethics, we searched seven electronic databases: PubMed, Ovid MEDLINE, Scopus, Philosopher’s Index, Embase and Web of Science. The database search was performed in November 2019 and included all relevant literature published up to that date (see Table 2). Language restriction was applied to the results, thus excluding studies not available in English. A total number of 3267 records was retrieved through the queries.

**Table 1:** Extended search query used in each database

Database	Group A: clinical ethics committees		Group B: evaluation
Philosopher's index	(noft(clinical ethics committee) OR noft(hospital ethics committee) OR noft(clinical ethics committees) OR noft(hospital ethics committees))	AND	(noft(assessment) OR noft(evaluation) OR noft(impact) OR noft(effectiveness) OR noft(assessments) OR noft(evaluations) OR noft(impacts))
Embase	Clinical ethics committee': ti, ab, kw OR ' clinical ethics committees': ti, ab, kw OR 'hospital ethics committee': ti, ab, kw OR 'hospital ethics committees': ti, ab, kw	AND	Evaluation: ti, ab, kw OR evaluations: ti, ab, kw OR 'assessment'/exp OR assessment: ti, ab, kw OR assessments: ti, ab, kw OR impact: ti, ab, kw OR impacts: ti, ab, kw OR effectiveness: ti, ab, kw)
PubMed	(Clinical ethics committees[MeSH major topic])	AND	((((((evaluation[Title/Abstract]) OR evaluations[Title/Abstract]) OR assessment[Title/Abstract]) OR assessments[Title/Abstract]) OR impact[Title/Abstract]) OR impacts[Title/Abstract]) OR effectiveness[Title/Abstract]
Medline	ETHICS COMMITTEES, CLINICAL.mp.[mp = tx, bt, ti, ab, ct, ot, nm, hw, fx, kf, ox, px, rx, ui, sy]	AND	evaluation.mp.[mp = tx, bt, ti, ab, ct, ot, nm, hw, fx, kf, ox, px, rx, ui, sy] OR evaluations.mp.[mp = tx, bt, ti, ab, ct, ot, nm, hw, fx, kf, ox, px, rx, ui, sy] OR assessment.mp.[mp = tx, bt, ti, ab, ct, ot, nm, hw, fx, kf, oz\x, px, rx, ui, sy] OR assessments.mp.[mp = tx, bt, ti, ab, ct, ot, nm, hw, fx, kf, ox, px, rx, ui, sy] OR impact.mp.[mp = tx, bt, ti, ab, ct, ot, nm, hw, fx, kf, ox, px, rx, ui, sy] OR impacts.mp.[mp = tx, bt, ti, ab, ct, ot, nm, hw, fx, kf, ox, px, rx, ui, sy] OR effectiveness.mp.[mp = tx, bt, ti, ab, ct, ot, nm, hw, fx, kf, ox, px, rx, ui, sy]
Web of science	TS = (clinical ethics committee* OR hospital ethics committee*)	AND	TS = (assessment* OR evaluation* OR impact* OR effectiveness*)
Scopus	((TITLE-ABS-KEY("clinical ethics committee")	AND	TITLE-ABS-KEY(effectiveness) OR TITLE-ABS-KEY(evaluation) OR TITLE-ABS-KEY(impact) OR TITLE-ABS-KEY(assessment)))

**Table 2:** Number of records for each database

Database	Date of search	Number of results
Philosopher's index	29/11/2019	71
Embase	29/11/2019	230
PubMed	29/11/2019	132
Ovid medline	29/11/2019	660
Web of science	29/11/2019	2127
Scopus	29/11/2019	47
	Total	3267

The screening process was then carried out by the first (CC) and the second author (VS) according to the PRISMA guidelines (Moher et al. 2009) and managing citations and available texts with Mendeley software (version 1.19.4).

First, duplicates (363) were excluded. The first author (CC) screened the remaining titles, according to preset inclusion and exclusion criteria (see below). The abstract screening (115) was then performed by the first author (CC) and the second author (VS) independently, to ensure scientific and methodological rigorousness of the abstract selection. In 91.5% of the abstracts there was agreement between the two authors, but consensus was reached after discussing doubtful candidate abstracts. A screening of the full text of the remaining records (71) was then performed by first author (CC) and the second author (VS) independently. After this step, a total of 27 articles was included in the review process.

Bibliographies of relevant articles were examined and two additional articles that met the inclusion criteria were retrieved through reference manual searching and included.

Finally, a total of 29 studies was included in the review. All the articles included were considered of a sufficient quality, based on the peer review process and on the academic reputation of the journals*.*

**Fig. 1:**
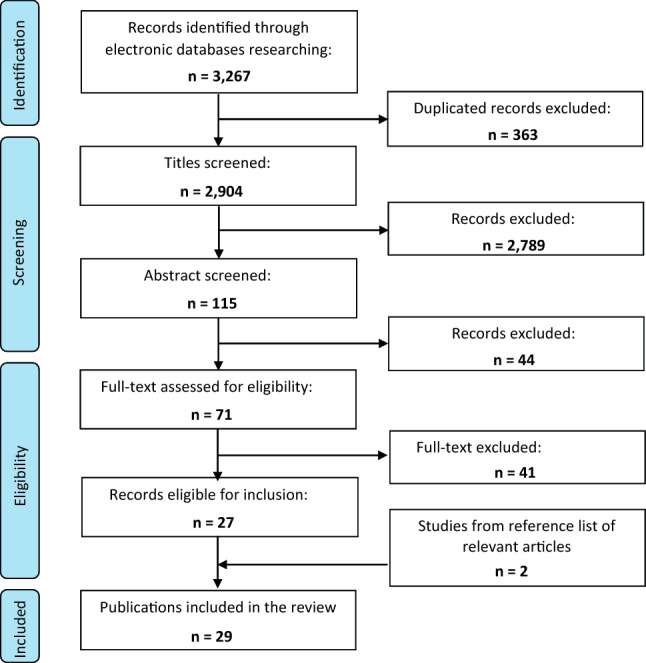
Flow chart showing the electronic databases search and articles selection procedure

The full process of selection is reported in the flow chart in Fig. 1.

### Inclusion criteria

Publications were included on the basis of both the following conditions: (a) address CECs as a specific topic and (b) provide an evaluation, assessment, impact of one or more aspects of CECs’ performance, whether *theoretically—*such as the description of an assessment model—or *empirically*, through quantitative and/or qualitative measures.

### Exclusion criteria

The following publications were excluded from the review: (a) studies addressing topics other than CECs as their main focus; (b) studies concerning CECs but not providing any form of evaluation, assessment, impact (e.g., describing CECs’ functions, without providing any assessment); articles (a) with no full text available (d) and not published in English, (e) editorials, books, and book chapters.

## Results

### General description of results

Twenty-seven articles from the research queries and two more papers identified through the snowball method met our inclusion criteria and became part of this systematic review (see Fig. 1) (Table 3).

**Table 3:** General description of the included studies

No.	Authors	Title	Country	Year of publication
1	Sullivan and Egan	A measure of growth	USA	1993
2	White et al.	A practical instrument to evaluate ethics consultation	USA	1997
3	Smith et al.	A survey of awareness and effectiveness of bioethics resources	USA	1992
4	White et al.	An account of the use fullness of a pilot clinical ethics programme at a Community Hospital	USA	1993
5	Day et al.	An assessment of a formal ethics committee consultation process	USA	1994
6	Førde et al.	Clinicians' evaluation of clinical ethics consultations in Norway: a qualitative study	Norway	2008
7	Scheirton	Determinants of hospital ethics committee success	USA	1992
8	Shetach	Dilemmas of ethics committee's effectiveness	Israel	2012
9	Bahus and Førde	Discussing end-of-life decisions in a clinical ethics committee: an interview study of Norwegian doctors' experience	Norway	2016
10	Hern	Ethics and human values committee survey: (AMI Denver Hospitals: Saint Luke's, Presbyterian Denver, Presbyterian Aurora: Summer 1989). A study of physician attitudes and perceptions of a hospital ethics committee	USA	1990
11	Storch et al.	Ethics committees in Canadian Hospitals: report of the 1989 survey	Canada	1990
12	Storch and Griener	Ethics committees in Canadian Hospitals: report of the 1990 pilot study	Canada	1992
13	Povar	Evaluating Ethics Committees: What do we Mean by Success?	USA	1991
14	Jansen et al.	Evaluation of a paediatric clinical ethics service	Australia	2018
15	Førde and Pedersen	Evaluation of case consultations in clinical ethics committees	Norway	2012
16	Hernando Robles	Evaluation of healthcare ethics committees: the experience of an HEC in Spain	Spain	1999
17	Gaudine et al.	Evolution of hospital clinical ethics committees in Canada	Canada	2010
18	Moeller et al.	Functions and outcomes of a clinical medical ethics committee: a review of 100 consults	USA	2012
19	Wilson et al.	HECs: are they evaluating their performance?	USA, Puerto Rico, Columbia, Canada	1993
20	Perkins and Saathoff	Impact of medical ethics consultations on physicians: an exploratory study	USA	1988
21	Magelssen et al.	Importance of systematic deliberation and stakeholder presence: a national study of clinical ethics committees	Norway	2019
22	Cohen	Interdisciplinary consultation on the care of the critically ill and dying: the role of one hospital ethics committee	USA	1982
23	Scheirton	Measuring hospital ethics committee success	USA	1993
24	Frolic et al.	Opening the black box of ethics policy work: evaluating a covert practice	USA	2012
25	Schochow et al.	The application of standards and recommendations to clinical ethics consultation in practice: an evaluation at German Hospitals	Germany	2017
26	Hauschildt et al.	The use of an online comment system in clinical ethics consultation	USA	2017
27	Pedersen et al.	What is happening during case deliberations in clinical ethics committees? A pilot study	Norway	2009
28	Ramsauer and Frewer	Clinical ethics committees and pediatrics: an evaluation of case consultation	Germany	2009
29	Orr et al.	Evaluation of an ethics consultation service: patient and family perspective	USA	1996

Publication dates range from 1982 to 2019, with five articles published in the last 5 years (9, 14, 21, 25, and 26). Of the twenty-nine articles included in the review, 23 made an evaluation based on data collected through empirical research and/or on the documents drafted by CECs’ members, such as the reports of meetings and discussions (1, 3–7, 9–12, 14, 15, 17, 18, 20–23, 25–29). The remaining six articles describe theoretical models for CECs’ evaluation (2, 8, 13, 16, 19, and 24). Amongst the latter, two articles also provide empirical data in support of (2) and/or to test (19) such model. It should be noted that two articles included in the review refer to the same study (7, 23). However, since they report different aspects of the same study, respectively the theoretical (7) and empirical (23) part of an assessment model for CECs’ effectiveness, we decided to include both publications.

The tools used for CECs’ evaluation were the followings: surveys only (2, 3, 4, 5, 7, 11, 14, 17, 19, 21, 23, and 25), interviews only (6, 9, 10, 12, 20, and 27), survey plus interviews (1, 29), survey plus anecdotal evidence (22). In addition, the authors of three studies made qualitative analyses on reports from case consultation (15, 18, and 26), or used medical charts to compare data from surveys and interviews (18, 20). The assessment tools are outlined in detail in Table 4.

**Table 4:** Details on evaluation and terminology

No.	Evaluated function	Measured aspects	Evaluation tool/s	Terms indicating effectiveness (recurrence)
1	CEC in general	Perceived helpfulness; satisfaction	Survey + interviews	Effectiveness (1); Performance (2); Usefulness (1)
2	Ethics consultation	Satisfaction, change in knowledge, in patient care, in length of stay and in intensity of treatment	Survey	Helpfulness (1); Performance (9); Success (1)
3	Ethics consultation	Perceived helpfulness	Survey	Effectiveness (5); Helpfulness (1)
4	CEC in general	Perceived effectiveness; satisfaction	Survey	Effectiveness (5); Impact (2); Usefulness (2)
5	Ethics consultation	Perceived effectiveness	Survey	Effectiveness (19); Helpfulness (1)
6	Ethics consultation	Perceived usefulness	Semi-structured interviews	Impact (1); Success (1); Usefulness (3)
7	CEC in general	Satisfaction	Survey	Effectiveness (1); Success (46)
8	CEC in general	Mission clarification; CEC authority; composition; appropriate set of regulations and procedures; efficient follow-up procedures	(not applicable)	Effectiveness (19); Efficiency (5); Performance (2); Success (3)
9	Ethics consultation	Satisfaction	Semi-structured interviews	Helpfulness (2); Usefulness (2)
10	Ethics consultation	Perceived effectiveness; satisfaction	Semi-structured interviews	Effectiveness (3)
11	CEC in general	Perceived effectiveness	Survey	Effectiveness (7); Impact (1); Usefulness (1)
12	CEC in general	Perceived effectiveness	Interviews	Effectiveness (4); Helpfulness (4)
13	CEC in general	Institutional acceptance; consensus within CEC’s members	(Not applicable)	Impact (1); Success (38); Usefulness (1)
14	Ethics consultation	Perceived helpfulness	Survey	Efficacy (7); Helpfulness (6); Usefulness (1)
15	Ethics consultation	Adoption of systematic approach	Qualitative analysis of reports	Usefulness (1)
16	CEC in general	CEC transparency; method of case deliberation; members’ participation in discussions; n. of recommendations followed; degree of compliance with accreditation rules	(Not applicable)	Effectiveness (2); Helpfulness (1); Success (4); Usefulness (2)
17	CEC in general	Perceived effectiveness; satisfaction	Survey	Effectiveness (16); Impact (13); Success (1)
18	Ethics consultation	No days required to complete consult; percentage of recommendations followed; patient outcome	Descriptive statistics on data + qualitative analysis on case reports	Efficacy (1); Impact (2); Success (1); Usefulness (1)
19	CEC in general	Extent of CECs self-evaluation	Survey	Effectiveness (9); Efficacy (1); Efficiency (1); Performance (4); Success (4)
20	Ethics consultation	Perceived impact; change in patient management	Semi-structured interview + revision of medical charts	Impact (14); Success (1); Usefulness (1)
21	Ethics consultation	Satisfaction	Survey	Helpfulness (9); Success (2); Usefulness (10)
22	Ethics consultation Policy development	Perceived impact	Survey + anecdotal evidence	Effectiveness (1); Impact (1);
23	CEC in general	No of developed guidelines; no of requests for educational programs; no of requests for case consultation; change in patient management	Survey	Effectiveness (11); Impact (5); Success (49); Usefulness (2)
24	Policy development	Perceived impact	Anecdotal evidence	Effectiveness (2); Efficiency (1); Helpfulness (5); Impact (3); Success (16); Usefulness (4)
25	Ethics consultation	Adherence to international standards	Survey	Impact (1)
26	Ethics consultation	Efficacy of the adoption of an online system for case discussion	Analysis of reports	Impact (2); Performance (1); Success (1)
27	Ethics consultation	Method of deliberation	Observation + Semi-structured interviews	Impact (1)
28	Ethics consultation	Usefulness; satisfaction; time spent on discussion	Qualitative analysis on reports	Performance (1); Usefulness (2)
29	CEC in general	Perceived helpfulness; change in patient management	Survey	Helpfulness (48); Efficacy (1)

The enrolled participants are physicians in twelve studies (41.4%) (2–6, 9, 10, 14, 20–22, and 24), CECs’ members in eight studies (26.6%) (1, 7, 17, 19, 21, 23, 27, 28), and the category of those who requested CEC’s intervention or were somewhat involved in the CEC’ processes, mainly as part of the attending healthcare team, in nine studies (30%) (2, 5, 6, 9, 12, 14, 20, 21, and 23). Patients and their families who took part in ethics consultation were invited to participate only in 10% of studies, in which they were asked to provide comments about the ethics services offered by the CEC (2, 5, and 29). In two studies, the composition of the sample is not clear, as the identity of respondents is not specified (11, 25). There was no sample in the three studies analyzing reports from case consultation (15, 18, and 26) and in the four theoretical studies (8, 13, 16, and 24).

### Function subjected to evaluation

Of the three functions that are typically attributed to CECs—ethics consultation, bioethics training, and revision and/or development of ethics policies—the mostly evaluated is ethics consultation, being the only subject assessed in sixteen studies (55.2%) (2, 4–6, 9, 10, 14, 15, 18, 20, 21, 25–29). This function may be performed in different ways, often in relation to the context in which the CEC is located (Boniolo and Sanchini 2016; Fournier 2015; Linkeviciute and Sanchini 2016). The predominant expression, according to our review, is “*ethics/clinical ethics consultation”* (2, 4, 9, 14, 17, 18, 20, 21, 25–29). The same can also be labeled as “*case consultation”* (3, 5, 6, 10, 12, 13, 15, 16, 18, 23, and 24), prospective and retrospective “*case review”* (7, 11), “*discussion forum”* (19), and “*case discussion”* (22).

We found that different conversation methodologies were used to carry out consultations (Linkeviciute and Sanchini 2016). This is in line with the fact that there is no unique mandatory procedure to perform them, though some countries proposed standards for ethics consultation (American Society for Bioethics & Humanities 2011). Among the methods described in this review, two are explicitly mentioned: the six-step model (15), a conversation methodology used to facilitate the research of possible solutions for an ethical issue, by outlining its elements and context (medical facts, involved parties, values at stake); the Nijmegen method (25), which applies relevant concepts from different normative ethical theories (such as hermeneutics and pragmatism) to case discussion (Kazeem 2014; Steinkamp and Gordijn 2003). In study 18, it is stated that the CEC choose which methodology to adopt depending on circumstances. With respect to remaining articles (2, 4–6, 9, 10, 14, 20, 21, 26–29), despite providing some insights on how CECs conducted ethics consultation, they do not specify which conversation methodology they were using, making it difficult to define whether they were following a specific methodology or adapting the consultation to the single case.

Of the other articles dealing with CECs’ functions, seven perform a general assessment of all three standard functions (1, 3, 7, 11, 12, 17, and 23) and one outlines a model to perform assessments (16).

Two studies propose a framework for measuring (13) and/or reaching (18) CEC’s success in all the three above-mentioned functions. Among the theoretical papers, one deals with the function of preparation and/or revision of ethics policies and provides a model for their successful development (24). The function of policy preparation and/or revision is also assessed, together with ethics consultation, in study 22.

The selection process did not identify studies focusing only on education and training in bioethics, though this is considered a core function of CECs (1, 11), with a positive impact for the healthcare staff (17).

Finally, study 19 investigates whether CECs carry out some kind of self-evaluation.

No selected article provides a comprehensive evaluation of CECs, looking at CECs’ functions separately.

### General findings

#### Terminological premises and review scope

The aim of the present work is to review the results of CECs’ assessments in order to clarify their effectiveness. To reach this aim, we systematically looked into the included articles in order to identify the exact expressions that refer to CECs’ evaluation. We found a variety of terms referring to CEC’s evaluation: effectiveness (1, 3, 5, 7, 8, 10–12, 17, 19, 23, 24), which is the most recurrent expression, efficacy (8, 14, 24), impact (6, 11, 13, 17, 18, 20, 23–27), success (2, 3, 7, 8, 13, 16–19, 23, 24), performance (1, 2, 19–21, 26), usefulness (1, 4, 6, 9, 16, 21, 23, 24, 28), helpfulness (2, 3, 5, 9, 12, 14, 21, 24, 29) (see Table 4). Note that, in many cases, even within the same article, these terms and expressions are used in an interchangeable manner, as synonyms, although they may have different connotations. In fact, the literature on the evaluation of CECs is heterogeneous and not only the *expressions* used to indicate CECs’ performance, but also the *meaning* of these expressions, as well as the *outcomes* considered as index of effectiveness, differ. In general, all the above mentioned terms may refer either to more objective outcomes, namely the tangible consequence of CECs’ activities on clinical practice (e.g., as a change in the management of patient care path), or to a more subjective outcome, namely, the experiences of the stakeholders—healthcare professionals, patients, and their families—who benefited from CECs’ services (e.g., satisfaction or perceived usefulness of the services). In this second meaning, CEC’s impact was measured mainly through questionnaires and/or semi-structured interviews.

The variety of both the expressions used in relation to CECs’ evaluation and their interpretation resulted in a variety of assessment tools employed as well as outcome observed in the selected articles: although we collected a reasonable number of articles about CECs’ evaluation, we were not able to find a standardized and unique measure for evaluating CECs’ efficacy. In those reported cases in which the same assessment criterion is used (e.g., satisfaction), neither there is a unique way for measuring it, nor it may be found a validated instrument justifying its use.

#### Subjective measures: users’ perception of CEC effectiveness

Most findings concern users’ perceptions. In particular, most studies investigate whether users and providers consider CEC’s activities, especially ethics consultation, to be helpful (1–6, 9, 10–12, 14, 20, 21, 22, and 29). Users are represented by physicians, staff members, residents or trainees (4, 6, 9, 10, 12, 20), nurses (4, 12), members of the healthcare team in general (2, 3, 5, 14, 21, 22), or professionals working within the hospital, such as social workers and pastoral care staff (3). Patients and their families are also included as users of the consultancy service, but only in a minority of studies (2, 5, and 29).

Despite potentially raising conflicts of interests, in some cases the evaluation of CEC’s performance is provided by hospital administrators (11, 12) and CEC’s own members, who are asked to assess how they perceive the impact of their own consultation activities (1, 4, 7, 17, 19, 21, and 23).

Satisfaction and positive overall judgment towards ethics consultation prevail over dissatisfaction not only in all the studies involving CEC’s members, as expected (1, 4, 7, 17, 19, 21, 23), but also when they involve users, with only one study reporting a higher percentage (66,6%) of physicians’ negative impression (10). Although data reported by the studies and tools used to collect them are too diverse to enable real comparisons, there seems to be a difference in users’ reported satisfaction levels. For instance, patients’ families or surrogates (i.e., layers, guardians, or friends) express appreciably lower average scores in satisfaction than the other groups of respondents. In fact, they rate ethics consultation helpful in 57% of the cases, according to study 29, and two out of six participants (33.3%) of study 5 claim they were very dissatisfied with the consultancy. On the other hand, according to the studies reporting percentages, perceived helpfulness ranges from 65% (4) to 96% (3) for healthcare professionals and from 81% (1) to 88% (4) for CEC’s members.

Among healthcare professionals, physicians seem the least satisfied category. In general, physicians are usually more critical towards different aspects of consultation services, even when they declare an overall satisfaction. They complain about the long response timelines to receive recommendations about cases submitted (9, 21), the lack of any systematic structure, improper analyses (9), and biases in case discussions (21). Physicians also express concerns about the composition of CECs, by which the presence of specialized professionals, or key figures whose presence during consultation sessions is essential for the completeness of the case discussion, should be increased (5, 6). In this view, including an acceptable number of clinicians would also prevent CEC discussions from being too theoretical and far from the daily routine of clinical practice (10). Other physicians raise doubts over CEC members’ expertise on matters discussed (9, 10) as well as on consultations’ real usefulness, questioning their need (12) and their effectiveness (10).

Differently, in all the studies in which they were enrolled, nurses appear as more satisfied than physicians, especially in relation to ethics consultation: although they seem to have less awareness and access to the ethics consultation services, 83% of nurses rate it as effective, in comparison to 65% of physicians (4, 12).

Although a unique and standardized tool for measuring CECs’ effectiveness was not found, articles selected provide relevant data on the impact of CECs’ activities, which may help in shedding some light on this topic. In more than one article, ethics consultation is considered to strengthen the decisions regarding patient management and to support physicians in their treatment intentions (4, 9, 10, and 20). Many physicians also report they learnt how to fruitfully discuss ethically sensitive issues from case consultations (6, 20, and 21). Other studies find the process of ethics consultation useful to improve quality of care (3) and to promote care values, even, in some cases, by helping hospitals to preserve their (religious) identity (12).

Other authors report a positive correlation between the degree of clinicians (2, 20) and/or patients’ families (29) satisfaction with respect to ethics service and the change in patient’s treatment, perceived as a positive result of the ethics consultation process. Remarkably, some changes in treatment plan occurred in thirty-one out of fifty-nine patients in study 20 and in 33% of patients in study 29.

Meetings devoted to ethics consultation are also considered as helpful opportunities to discuss ethically relevant issues (6, 9, 11, and 20), insofar as they are also able to provide healthcare professionals and patients’ families with emotional and social support (4). This evidence is also supported by studies showing a correlation between ethics consultation and a decrease in the level of distress among hospital staff members (14), and among patients and their surrogates (29). In paper 14, twenty-eight out of the thirty-five healthcare professionals involved in the study reported a decrease in moral distress due to consulting ethics services, while in study 29, patients and their surrogates declare that ethics consultation was “reassuring”, “supportive”, and “took the weight off” their shoulders (29, p. 137). In general, ethics consultation may give a voice to all the individuals facing—albeit differently—ethical issues in clinical practice, thus making physicians, patients and their families feel that their concerns and perspectives matter (6, 39).

#### Objective measures of CEC effectiveness

More objective evaluation measures include qualitative analyses of ethics consultation reports, with the aim of evaluating how CECs work during case deliberation and/or how case discussion is conducted (15, 18, 26–28). These studies also report, when available, the number of cases in which CECs’ suggestions have been then actually followed by relevant players (18), as well as the following information: the reason for requesting the consultation (18), whether ethical issues have been correctly identified and analyzed, by what method (15, 26), if the discussion takes place following a specific structure or set of steps (15), how much time is dedicated to the meeting (27, 29), and how much time is needed to provide requesters with a response (18, 29).

Considerations resulting from the theoretical articles are in line with the aforementioned empirical data. More than one article underlines the importance of multidisciplinarity, encouraging CEC to be composed in such a way so as to incorporate all relevant expertise and disciplines (8, 13, 16). They also highlight the importance of having systematic discussions during CEC’s meetings (8, 16). Another point is the concept of *meaningful consensus* as a criterion for successfully delivering ethics consultations (13). With respect to the latter, the idea was raised that consensus among CEC’s members in case discussion is not necessarily a value per se, as it could be due to a lack of divergent views or the dominance of a single committee member.

## Discussion

This review shows that CECs seem to exert a positive impact both on the healthcare personnel and the institutions in which they work, but many aspects of their functioning are still left to dissect. It is apparent that there is a great diversity in the procedures they adopt, mostly in relation to their cultural and geographical contexts. This also makes it difficult to get to shared criteria for their evaluation.

Heterogeneity in assessments raises methodological difficulties to make straightforward comparisons and to identify the key factors for a positive impact. Criteria by which CECs’ activity is considered successful, and the definition of success itself, varies considerably from study to study, and from context to context. This makes it difficult to evaluate CECs’ performance. Therefore, the adoption of clear (and, as much as possible, shared) standards would be useful. However, cultural diversities should be also respected. CECs are meant to be so close to clinical practice that a globally harmonized metric of their success may be unconceivable and possibly not desirable. Nonetheless, as a matter of fact, CECs—particularly in regard to their function of ethics consultation—were largely reported as beneficial by both users and providers in many studies.

Clearly, ethical consultation is perceived as the main core business of CECs. Unfortunately, assessing its efficacy is problematic (Hoffmann 1993; Linkeviciute et al. 2016). There is no consensus about which tools to use (Ramsauer and Frewer 2009). Most studies adopted satisfaction as a measure of effectiveness. However, satisfaction and/or perceived helpfulness are obviously subjective criteria and, as such, depend on multiple variables that are not always quantifiable or reliable. In any case, it is more than reasonable that users’ satisfaction may be a tool, if properly thematized. Delany and Hall provide a broad view of satisfaction, which combines empowerment, enhanced understanding and the feeling of being more prepared to face some conditions (Delany and Hall 2012). Following this concept, satisfaction would be determined by an increased understanding of ethical issues and moral values at stake, thanks to multidisciplinary discussions and ethical analyses during case discussions, with a willingness to follow insights and recommendations as a result. In the end, with regard to the primary objective of CECs—namely, to provide support to healthcare professionals on clinical cases—satisfaction may well be a reasonable performance indicator. The decreased level of distress, reported as a result of ethics consultations, also seems to indicate successful support of healthcare professionals, at least at an emotional level. Although not widely reported, it is important to underline that some studies mention changes in patient management and therapeutic plans as a consequence of ethics consultation.

Albeit few studies have investigated this aspect and more research is needed, this finding could indicate how a broadening of perspectives as allowed by the ethical multidisciplinary review can affect the decision-making process and impact on clinical decisions, thus improving the quality of patient care (Gorini et al. 2012; Kondylakis et al. 2017). To ensure that this is the case, the composition of committees should include as much expertise as possible in the relevant areas of ethical-clinical issues that are addressed, including experts in ethics and bioethics (Sanchini 2015), to maximize multidisciplinarity (Gilardi et al. 2014).

In regard to the educational function, the lack of studies thereon is worth mentioning. In our review, although several authors stress its importance (Storch et al. 1990; Sullivan and Egan 1993), bioethics training seems to be underestimated or underreported. Indeed, amongst the three functions of CECs, this should be the easiest to assess. In addition, its impact should almost be a given: by being properly trained, healthcare professionals will inevitably become more sensitive to ethical issues, and potential ethical threats may be prevented. The possible lack of resources allocated to bioethical training, as compared to those devoted to ethics consultation, would suggest that CECs see ethics consultation as their main task (Ramsauer and Frewer 2009). This is not surprising assuming that CECs were originally established to support healthcare professionals in facing and managing ethical issues involved in clinical practice. This function is therefore perceived as the main one, and the most tangible, with respect to the other functions, albeit considered helpful and worthy (Smith et al. 1992). On the other hand, one may observe that the most effective way to train physicians about bioethical issues is likely through real clinical case discussions (Førde et al. 2008; Magelssen et al. 2019; Perkins and Saathoff 1988). Thus, the function of ethics consultation could actually imply an educational added value as a kind of “by-product”, in a way which could be less theoretical and more palatable to clinicians than more conventional training strategies. Of course, it should be noted that this “field training” would be less accessible than “class training” and limited to those who require the support of CECs, namely those who in some way are already prone to recognize the ethically problematic aspects of a clinical case and are willing to discuss it.

In regard to the function of working out and reviewing institutional policies, any attempt to evaluate its impact is difficult. Indeed, whatever the processes of drafting these institutional guidelines, how much they actually affect clinical practice is an open issue. Investigating this item is challenging, in the end as much as it has always been challenging in clinical medicine to assess the impact of clinical practice guidelines. Probably, however, an outstanding added value of guidelines in general is the process of their preparation, as long as it involves many clinicians and leads them to be aware of, and discuss, issues which may often be underappreciated or ignored. In this sense, it is more than likely that CECs may expose clinicians and health administrators to a multidisciplinary array of skills and perspectives which otherwise could be missed.

One last observation based on publication dates and the geographical distribution of the studies we reviewed seems to indicate a decrease over the last years in the number of articles about CECs’ functions and activities in the United States, where nowadays they are viewed as being routinely a component of healthcare institutions. In the US, CECs’ presence in hospitals and healthcare institutions may be so deeply rooted that investigating their effectiveness may not seem an interesting matter anymore. On the other hand, the interest in CECs is on the rise in Europe, where CECs are still developing (Bahus and Førde 2016; Magelssen et al. 2019; Schochow et al. 2017).

### Quality of selected studies

All the 29 selected articles were considered of sufficient quality for inclusion in the present review. However, quality varies from article to article, depending on how studies were designed and carried out, as well as on the comprehensiveness of data. Therefore, while for the theoretical articles providing models of evaluation we considered sufficient the quality criteria listed in *Methods* (reliability of peer-review processes and academic reputation of the journals), we proposed a quality assessment (from low to high) for the ones reporting empirical data. Data considered for quality assessment were the followings: the type of evaluation tool employed, whether the complete dataset was reported, the number and description of enrolled subjects or the number of documents analyzed, and the response rate. We excluded potentially interesting papers (i.e. papers that could have met our inclusion criteria) if they showed a low quality, according to our assessment criteria (Table 5).

**Table 5:** Quality of the studies included in the systematic review

No.	Measure of evaluation	Evaluation tool attached to the paper		Comprehensive response dataset		Sample size	Sample description	Response rate	Evaluation rate of the papers included		
		Yes	No	Yes	No				Low	Medium	High
1	Review of minutes of CECs' meetings, survey, semi-structured interviews		×		×	21 (survey) 14 (interviews)	CEC's members (current and former)	81% (survey) 66% (oral evaluation)	×		
2	Survey	×		×		45	Consultation requestor (healthcare professionals, patients and families)	60%			×
3	Survey	×		×		1957	Staff and resident physicians, nurses, social workers, pastoral care staff	41%			×
4	3 surveys (pre-test, post-test + follow up)		×	×		157 (pre-test) 168 (post-test)	Nurses, physicians of critical and special care units (pre-test and post-test) Ethicists, nurses, physicians (follow-up)	29/10% (nurses/physicians) (pre-test) 18/19% (nurses/physicians) (post-test); 98% ethicists, 54% nurses, 52% physicians (follow-up)		×	
5	Survey (2 versions: for healthcare staff and patient's family members	×		×		46	Healthcare staff (40) and patient's family members (6) involved in one of the 16 HEC consultations object of the evaluation	54% (staff members) 30% (family members)			×
6	Semi-structured interviews		×	×		8 (for 10 consultations)	Physicians (from 6 different hospitals)	50%		×	
7	Survey		×	×		137	HEC chairpersons of hospitals with > 100 medical residents	83%		×	
8	(Not applicable)					(Not applicable)	(not applicable)	(Not applicable)			
9	Semi-structured interviews	×		×		15	Physicians who requested CEC consultation	–			×
10	Semi-structured interviews	×			×	10	Physicians who admitted at least 25 patients at local hospital in the previous 12 months	25% (met inclusion criteria)		×	
11	Survey		×		×	72	Respondents from English language hospitals with > 300 beds	84.5%	×		
12	Survey, Semi-structured interviews		×		×	?	Staff from 5 hospitals with a CEC: physicians, nurses and administrators	–	×		
13	(Not applicable)					(Not applicable)	(Not applicable)	(Not applicable)			
14	Web-based survey	×		×		35 (11 cases surveyed)	Staff involved in a consultation: consultants physicians (16), nurses (10), allied healthcare professionals (4), trainee physicians (3), social workers (2)	36%		×	
15	Case discussion reports	×		×		17	(Not applicable)	46%			×
16	(Not applicable)					(Not applicable)	(Not applicable)	(Not applicable)			
17	Survey	×		×		107	Chairpersons of French and English-language hospitals with a CEC and > 100 beds	51%			×
18	Case discussion reports		×	×		100 (for 98 patients)	(Not applicable)	(Not applicable)		×	
19	Survey	×		×		236	CEC members of active and functioning CECs				×
20	Telephone semi-structured interview, revision of medical charts (to be compared with results from interviews)		×	×		34 physicians	Physicians requesting CEC consultations	94%		×	
21	Survey (2 versions: CEC members, clinicians), each to be replied in relation to a single consultation	×		×		64	CECs' members and clinicians who took part in CEC meetings	52.7% (participating CECs) 63% (reported consultations)			×
22	Informal survey, anecdotal evidence		×		×	(Not available)	Local hospital staff	(Not available)	×		
23	Survey		×	×		127	Chairperson of CECs from hospitals with > 100 beds and a major affiliation with a medical school	83%		×	
24	(Not applicable)					(Not applicable)	(Not applicable)	(Not applicable)			
25	Survey		×	×		545	Not available	29.3%		×	
26	Case discussion reports		×	×		159	CEC members	(Not applicable)		×	
27	Observations of committees deliberating a paper case (25 min) Semi-structured group interviews	×		×		9 CECS	CEC members				×
28	Case discussion reports		×		×	16	CEC ethicists	(Not applicable)	×		
29	Survey, semi-structured interviews		×	×		56	Patients or surrogates	90.3%		×	

### How assessing CECs’ effectiveness? Possible suggestions for CECs’ evaluation

Our comprehensive analysis may suggest some proposals to improve the way we can assess CECs’ effectiveness in regard to their three main functions.

With regard to the most widely evaluated function—*ethics consultation*—as many suggest, it is essential to assess whether and how ethical advice impacts on clinical decisions and their stakeholders. This means investigating whether and to what extent health professionals believe that ethics consultations improve patient care, and, specularly, whether and to what extent patients and their families believe that it resulted in a better and more comprehensive care process. We propose that the best way to maximize the amount of collected data and their exhaustiveness is to use both quantitative and qualitative methods. Indeed, questionnaires are the preferred methods to collect large amounts of data, for they facilitate researchers in reaching many people rapidly. On the other hand, qualitative methods, such as semi-structured interviews or focus groups, provide more extensive data, as they allow to deepen topics of interest and follow experiential flows. We also propose that consultation services should be monitored in the long run: given the specificity of ethical consultation and the low number of consultations per year (Hurst et al. 2005; Mino 2000; Slowther et al. 2001), data on a service collected longitudinally would be highly informative and would make it easier to intercept any potential impact of ethics consultation, for instance greater therapy compliance by patients, or less conflicts with families.

With respect to the *bioethics training* function, a comprehensive assessment should consider a twofold aspect. First, it should evaluate the acquisition of theoretical notions by using simple tests. As an example, to evaluate the effectiveness of a training session on the informed consent process, it should be assessed whether the trainee has learned the ethical pillars of a valid informed consent form and process (e.g., information, comprehension, voluntariness).

When training also aims to transfer operational skills (as stated by the American Society for Bioethics and Humanities), any assessment on the application of such skills should take into account that this is an ongoing and iterative process. The evaluation methods should also be modeled according to the specific skill/s conveyed, and on the audience it is addressed—namely, the hospital staff or the internal members of the CEC’s itself. As an example, in the first case, if the skills conveyed regard performing ethics consultation, the training sessions should teach the healthcare professionals first to recognize whether a case is ethically sensitive and then the key elements of ethics consultation (e.g. learning how to analyze, from an ethics standpoint, a clinical case, at least in a preliminary way); in this case, the assessment should require the trainee to apply the acquired skills, for instance by asking trainees to discuss an ad hoc clinical ethics case, recognizing the moral dilemma and analyzing the underlying ethical problem. Depending on the resources available, such an assessment can be made either through an oral test, or a focus group, or through a written examination.

Concerning the in-house training for CEC's members, as this is on-going training, the assessment should also be on-going. In this case, the members' skills to provide ethics consultation can be tested either through a test at the end of each course (e.g., by giving a case and verifying that they are able to analyze it); or through a training day in which this skill is updated and reinforced, for example by collecting particularly relevant cases and using them to practice moral case analysis. Again, the evaluation can be either oral or written.

With regard to the third function—*policy preparation and/or policy revision*—a key element to evaluate CECs’ performance is to verify if policies have been approved and enforced. Moreover, as it is always fundamental that healthcare professionals of a given institution develop an “ownership feeling” (Doyal 2001) with respect to policies affecting their practice, satisfaction questionnaires may be useful. However, it should be noted that this function is the most complicated to assess, because the implementation of any new or modified policy depends on many factors, such as, for example, administrative and organizational ones.

## Limitations

A limitation of our systematic research concerns the publication dates of studies included. Although we included five papers published within the last five years, more than half of the articles (16 studies) were written and published before the year 2000. Data reported by those studies would need an update. Only in one case, we noticed an update of data concerning the same CEC through the use of the same questionnaire (Gaudine et al. 2010; Storch et al. 1990).

## Conclusions

The aim of this systematic review was to provide an answer to the question whether CECs may be useful, by collecting all evaluation tools used by researchers to assess their impact in clinical practice. Although a definitive answer to this question cannot be provided, our work systematically collected available information. By doing this, our study provides a comprehensive overview of CECs’ impact, highlighting some key elements of their performance. Amongst the three most typical functions of CECs—namely, ethics consultation, policy formation and/or revision, and bioethics education—ethics consultation is largely overwhelming.

Despite the lack of standardized assessment tools, CECs appear to be beneficial at the very least in terms of healthcare professionals’ satisfaction. Indeed, the presence of CECs correlates with a lower moral distress among staff members.

However, this systematic review stresses the importance of developing standardized tools for evaluating ethics consultation. More work is needed to collect more data with respect to patients and/or their surrogates’ perspectives on this issue. Definitely, in view of an increasingly demand for personalized medicine, the patient’s perspective cannot be left aside.

## Data Availability

The authors declare that all the data supporting the findings of this study are available within the article.

## Code availability

No software application nor custom code was used.
